# Supplementary material for: Hot spot prediction in protein-protein interactions by an ensemble system
Source: BMC Syst Biol. 2018 Dec 31;12(Suppl 9):132. doi: 10.1186/s12918-018-0665-8 (PMC6311905; doi:10.1186/s12918-018-0665-8)
Supplement: Supplementary file 2 — All relevant MATLAB code. A simple Matlab implement of our predictor. (ZIP 19,742 kb) [file 12918_2018_665_MOESM2_ESM.zip › Additional file 2/Read ASA/ASA website/SKEMPI/1FFWB.htm]

 NetSurfP - Protein Surface Accessibility and Secondary Structure Predictions

  

|  |  |  |
| --- | --- | --- |
|  |  | NetSurfP - Protein Surface Accessibility and Secondary Structure Predictions  Technical University of Denmark |

  


---

```
# For publication of results, please cite:
# A generic method for assignment of reliability scores applied to solvent accessibility predictions.
# Bent Petersen, Thomas Nordahl Petersen, Pernille Andersen, Morten Nielsen and Claus Lundegaard
# BMC Structural Biology 2009, 9:51 doi:10.1186/1472-6807-9-51
#
# Column 1: Class assignment - B for buried or E for Exposed - Threshold: 25% exposure, but not based on RSA
# Column 2: Amino acid
# Column 3: Sequence name
# Column 4: Amino acid number
# Column 5: Relative Surface Accessibility - RSA
# Column 6: Absolute Surface Accessibility
# Column 7: Z-fit score for RSA prediction
# Column 8: Probability for Alpha-Helix
# Column 9: Probability for Beta-strand
# Column 10: Probability for Coil
E A  1FFWB                  1    0.882  97.141   0.432   0.003   0.003   0.994
E R  1FFWB                  2    0.563 128.904   0.225   0.021   0.279   0.699
E R  1FFWB                  3    0.470 107.653   1.270   0.023   0.655   0.322
B I  1FFWB                  4    0.115  21.368  -0.093   0.018   0.846   0.136
E I  1FFWB                  5    0.438  81.085   1.499   0.011   0.918   0.071
B L  1FFWB                  6    0.143  26.220   0.086   0.018   0.846   0.136
E S  1FFWB                  7    0.372  43.575   0.744   0.004   0.616   0.381
E R  1FFWB                  8    0.601 137.652   0.493   0.005   0.336   0.660
B L  1FFWB                  9    0.118  21.551   0.700   0.018   0.088   0.893
E K  1FFWB                 10    0.593 121.960   0.399   0.018   0.047   0.935
E A  1FFWB                 11    0.611  67.365   0.267   0.430   0.016   0.555
E G  1FFWB                 12    0.638  50.226   0.996   0.406   0.004   0.590
E E  1FFWB                 13    0.381  66.526   0.073   0.502   0.002   0.495
E V  1FFWB                 14    0.236  36.258   1.394   0.858   0.002   0.139
E D  1FFWB                 15    0.629  90.711   1.243   0.923   0.002   0.076
E L  1FFWB                 16    0.234  42.809   1.518   0.970   0.001   0.030
B L  1FFWB                 17    0.063  11.609   0.880   0.970   0.001   0.030
E E  1FFWB                 18    0.359  62.735   1.226   0.970   0.001   0.030
E E  1FFWB                 19    0.584 101.937   0.534   0.970   0.001   0.030
E E  1FFWB                 20    0.332  58.000  -0.091   0.923   0.002   0.076
B L  1FFWB                 21    0.137  25.066   0.019   0.782   0.003   0.216
E G  1FFWB                 22    0.469  36.934   0.206   0.502   0.002   0.495
E H  1FFWB                 23    0.459  83.438  -0.317   0.406   0.004   0.590
B L  1FFWB                 24    0.188  34.350   0.282   0.268   0.043   0.689
E T  1FFWB                 25    0.323  44.786  -1.176   0.113   0.087   0.800
E T  1FFWB                 26    0.539  74.787   1.234   0.066   0.296   0.638
B L  1FFWB                 27    0.136  24.865   0.427   0.074   0.484   0.442
E T  1FFWB                 28    0.438  60.709   0.370   0.074   0.484   0.442
E D  1FFWB                 29    0.439  63.217  -0.036   0.074   0.484   0.442
E V  1FFWB                 30    0.481  73.945   0.394   0.074   0.484   0.442
E V  1FFWB                 31    0.451  69.257   0.329   0.022   0.359   0.619
E K  1FFWB                 32    0.611 125.703   0.307   0.020   0.205   0.775
E G  1FFWB                 33    0.607  47.771  -0.344   0.018   0.047   0.935
E A  1FFWB                 34    0.578  63.740  -1.859   0.018   0.047   0.935
E D  1FFWB                 35    0.615  88.636  -0.778   0.005   0.045   0.951
E S  1FFWB                 36    0.486  56.959   0.699   0.004   0.514   0.481
B L  1FFWB                 37    0.138  25.341   0.507   0.002   0.816   0.182
E S  1FFWB                 38    0.383  44.876   0.694   0.001   0.959   0.040
B A  1FFWB                 39    0.114  12.563   0.311   0.001   0.959   0.040
B I  1FFWB                 40    0.258  47.804   0.868   0.001   0.959   0.040
B L  1FFWB                 41    0.150  27.410   0.926   0.001   0.959   0.040
E P  1FFWB                 42    0.473  67.076  -0.310   0.003   0.718   0.279
B G  1FFWB                 43    0.330  25.979  -0.913   0.005   0.336   0.660
E D  1FFWB                 44    0.640  92.210  -0.634   0.020   0.205   0.775
B I  1FFWB                 45    0.222  41.014   0.186   0.019   0.141   0.840
E A  1FFWB                 46    0.460  50.736   0.009   0.018   0.047   0.935
E E  1FFWB                 47    0.570  99.649  -0.500   0.268   0.043   0.689
E D  1FFWB                 48    0.609  87.728  -0.309   0.257   0.016   0.727
E D  1FFWB                 49    0.326  46.948   0.511   0.268   0.043   0.689
B I  1FFWB                 50    0.083  15.429   0.567   0.352   0.332   0.316
E T  1FFWB                 51    0.287  39.779   0.992   0.169   0.612   0.219
B A  1FFWB                 52    0.056   6.160   0.904   0.072   0.855   0.073
B V  1FFWB                 53    0.037   5.672   0.466   0.072   0.855   0.073
B L  1FFWB                 54    0.031   5.676   0.856   0.072   0.855   0.073
B C  1FFWB                 55    0.027   3.777   0.603   0.011   0.918   0.071
B F  1FFWB                 56    0.043   8.650   0.730   0.011   0.918   0.071
B V  1FFWB                 57    0.060   9.161   0.534   0.011   0.918   0.071
B I  1FFWB                 58    0.075  13.912   1.112   0.021   0.756   0.223
E E  1FFWB                 59    0.346  60.376   1.216   0.022   0.359   0.619
E A  1FFWB                 60    0.325  35.771  -0.184   0.052   0.084   0.864
E D  1FFWB                 61    0.605  87.253  -0.673   0.056   0.142   0.802
E Q  1FFWB                 62    0.372  66.457   0.487   0.069   0.386   0.545
B I  1FFWB                 63    0.142  26.233   0.775   0.023   0.655   0.322
E T  1FFWB                 64    0.406  56.340   0.426   0.018   0.846   0.136
B F  1FFWB                 65    0.226  45.438  -0.061   0.018   0.846   0.136
E E  1FFWB                 66    0.524  91.490   0.475   0.021   0.756   0.223
E T  1FFWB                 67    0.486  67.367  -0.132   0.022   0.552   0.426
E V  1FFWB                 68    0.655 100.597  -0.857   0.005   0.015   0.979

Explain the output.  Go back.
```

---
